# High-mobility capacitively-induced two-dimensional electrons in a lateral superlattice potential

**DOI:** 10.1038/srep20967

**Published:** 2016-02-11

**Authors:** T. M. Lu, D. Laroche, S.-H. Huang, Y. Chuang, J.-Y. Li, C. W. Liu

**Affiliations:** 1 Sandia National Laboratories, Albuquerque, New Mexico 87185, USA; 2 Department of Electrical Engineering and Graduate Institute of Electronic Engineering, National Taiwan University, Taipei 10617, Taiwan, R.O.C; 3 National Nano Device Laboratories, Hsinchu 30077, Taiwan, R.O.C

## Abstract

In the presence of a lateral periodic potential modulation, two-dimensional electrons may exhibit interesting phenomena, such as a graphene-like energy-momentum dispersion, Bloch oscillations, or the Hofstadter butterfly band structure. To create a sufficiently strong potential modulation using conventional semiconductor heterostructures, aggressive device processing is often required, unfortunately resulting in strong disorder that masks the sought-after effects. Here, we report a novel fabrication process flow for imposing a strong lateral potential modulation onto a capacitively induced two-dimensional electron system, while preserving the host material quality. Using this process flow, the electron density in a patterned Si/SiGe heterostructure can be tuned over a wide range, from 4.4 × 10^10^ cm^−2^ to 1.8 × 10^11^ cm^−2^, with a peak mobility of 6.4 × 10^5^ cm^2^/V·s. The wide density tunability and high electron mobility allow us to observe sequential emergence of commensurability oscillations as the density, the mobility, and in turn the mean free path, increase. Magnetic-field-periodic quantum oscillations associated with various closed orbits also emerge sequentially with increasing density. We show that, from the density dependence of the quantum oscillations, one can directly extract the steepness of the imposed superlattice potential. This result is then compared to a conventional lateral superlattice model potential.

In the past few decades, there has been much interest in the physics of two-dimensional electron gases (2DEGs) in a lateral superlattice potential. Such systems were the platform for understanding and demonstrating the Hofstadter’s butterfly^1^,^2^,^3^,^4^,^5^, quantum chaos in an antidot superlattice potential^6^,^7^,^8^, composite fermions at the Landau level filling factor 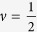 9, and have been proposed to engineer artificial graphene^10^,^11^,^12^,^13^,^14^,^15^,^16^, Bloch oscillators^17^,^18^,^19^,^20^, and semiconductor qubits^21^,^22^.

Aside from single-layer materials^4^,^5^, the conventional starting point for building these lateral superlattice systems is modulation-doped semiconductor heterostructures, in which electrons transfer from a doped electron-supply layer to a quantum well and form a 2DEG^23^. While electrons in GaAs/AlGaAs heterostructures have been the main platform^2^,^3^,^6^,^9^,^24^,^25^,^26^,^27^,^28^,^29^,^30^,^31^,^32^ due to the mature material growth technology, other material systems, including Si/SiGe^33^,^34^, Ge/SiGe^35^, InAs/GaSb^36^, and AlAs/AlGaAs^37^, have been explored. Using various nano-patterning tools such as e-beam lithography^3^,^6^,^9^,^28^,^29^,^36^, focused ion-beam^24^, local oxidation with atomic force microscope^30^,^31^, interference lithography^25^,^26^,^33^,^35^, in combination with wet/dry etching^6^,^9^,^28^,^33^,^35^,^36^, ion implantation^26^, and/or metal deposition^3^,^25^,^29^,^30^,^31^, a superlattice potential can be imposed onto the underlying 2DEG. To impose a sufficiently strong potential, etching through the doped electron-supply layer is usually required. This etching, however, significantly damages the host material, and, as such, degrades the electron mobility, or equivalently the electron mean free path. A shortened mean free path masks effects arising from large electron orbits in a magnetic field, and also leads to a reduced phase coherence length, making band structure engineering challenging. One metric for this degradation in material quality is the ratio of zero-magnetic-field mobilities before and after patterning (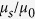), which is typically of the order of 10–100 for deep etched devices^6^,^9^,^28^,^32^,^33^,^35^. Another limitation encountered when using doped heterostructures to fabricate superlattices is the limited density range. Indeed, it has been shown that density tuning using a capacitively coupled gate is mostly ineffective in such doped devices^3^,^29^. A popular alternative is to change the electron density through persistent photoconductivity^3^,^6^,^24^,^32^,^33^. However, this method is irreversible until a thermal cycling of the device is performed, and the density tuning is not precise.

Alternatively, undoped semiconductor heterostructures, in particular GaAs/AlGaAs^38^,^39^,^40^ and Si/SiGe^41^ systems, have been demonstrated to host high-mobility 2D electrons and holes with a wide tunable density range. Instead of doping the host material, a gate is used to capacitively induce carriers in the quantum well. Such an enhancement-mode heterostructure field-effect transistor architecture has in fact been used in studies of 2D electron physics^42^,^43^,^44^ and engineered to form semiconductor quantum dots for quantum computation^45^,^46^. In the following, we present a fabrication process flow for building a capacitively induced 2DEG in a lateral superlattice potential. We demonstrate that the device hosts a 2DEG with a wide tunable density range with limited mobility degradation. We observe commensurability oscillations in the magneto-resistance, which arise from semi-classical cyclotron motions encircling one or several antidots. With increasing density and mean free path, contributions from increasingly large orbits appear sequentially. We also observe sequential emergence of quantum oscillations periodic in magnetic field. From the density dependence of these oscillations, we directly extract the steepness of the imposed superlattice potential, and compare the results to a conventional antidot superlattice model potential. Such a direct measure of the superlattice potential was not achieved in previous studies, where the steepness of the potential was only inferred through numerical simulations reproducing the experimental data *a posteriori*^6^,^47^.

## Results

### Superlattice patterning

**Figure 1:**
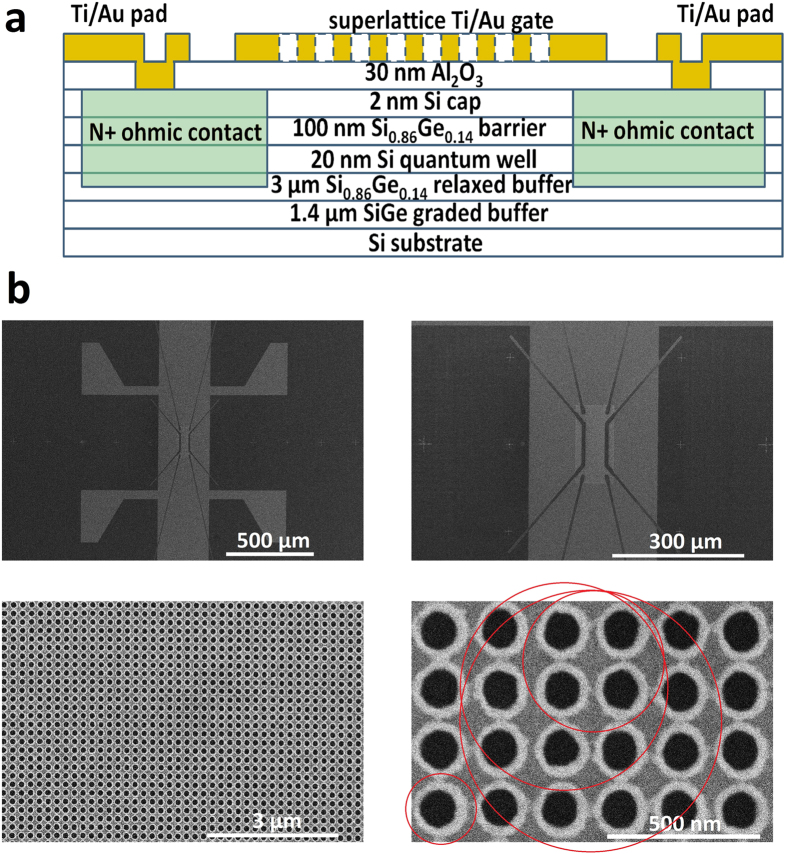
(**a**) A schematic drawing of the cross section of the device. (**b**) SEM images of the top view of the device. Upper panels: the active Hall bar defined in a larger Hall bar by 6-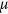
m-wide trenches. Lower panels: Zoom-in views of the superlattice area. The period of the hole array is 200 nm, and the diameter of the holes is 110 nm. The red circles represent cyclotron orbits encircling 1, 4, 9, and 16 antidots deduced from magneto-resistance peaks.

The starting material used in this study is a standard undoped Si/SiGe quantum well heterostructure grown in a ultra-high-vacuum chemical-vapor-deposition system (see Methods). Transport properties of induced 2D electrons in an un-patterned device from this material have been reported elsewhere^48^. A hole array with a period *d* = 200 nm in both directions is defined over a 90 *μ*m × 180 *μ*m region by locally ion milling the top gate (see Methods). Fig. 1a shows a schematic drawing of the cross section of the device. The device operates in enhancement-mode, where a positive gate bias greater than the threshold voltage capacitively induces electrons in the Si quantum well. Since the gate contains a hole array, the area under each hole is of higher potential for electrons, effectively constituting a lateral superlattice potential. In Fig. 1b we display four scanning-electron-micrographs of the top view of the device. The images show that the width of the active Hall bar is 44 *μ*m, the spacing between the two longitudinal voltage probes is 2.7 squares, the period of the square superlattice is ~200 nm in both directions, and the holes are circular with a diameter of ~110 nm.

### Magneto-transport characterization

We performed magneto-transport measurements at *T* = 0.3 K and obtained the longitudinal resistance (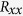), longitudinal resistivity (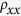), and the Hall resistance (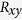) of the device (See Methods). The electron density can be extracted from low-field 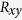 (*n*′) and also from high-field Shubnikov-de Haas oscillations (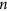). We use 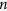 as the relevant electron density throughout this work, as it has been shown that significant deviation in 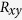 from the ideal behavior could occur in a 2DEG with a superlattice potential^6^,^47^. Deviations of *n*′ from 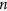 are indeed observed in our system (See Supplementary Information).

**Figure 2:**
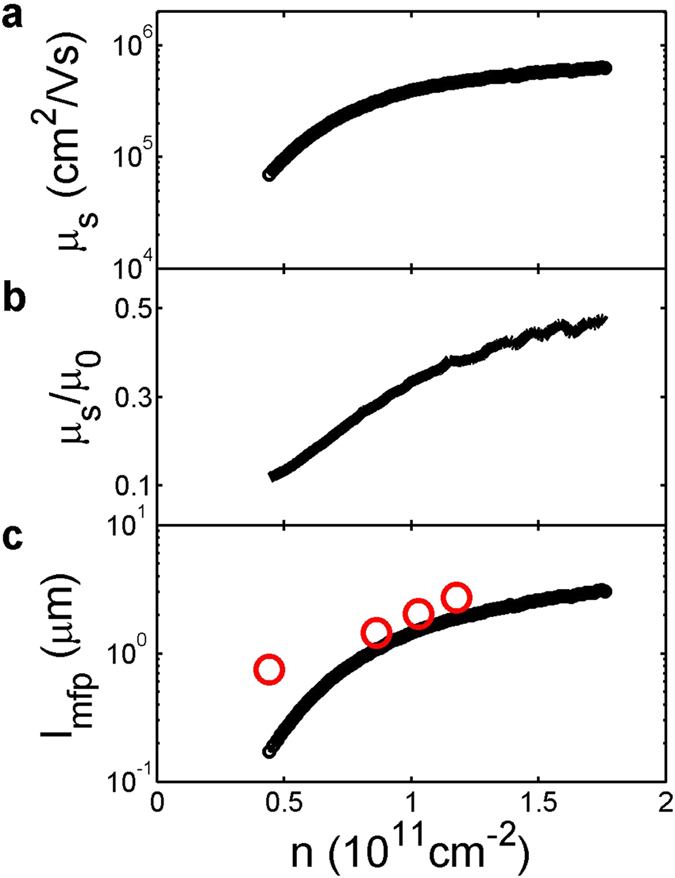
(**a**) Electron mobility 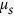 as a function of electron density 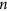 for the superlattice sample at *T* = 0.3 K. (**b**) The ratio of mobilities of the superlattice sample and an un-patterned sample (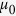) versus density 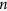. (**c**) The mean free path 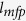 for the superlattice sample calculated from the mobility curve. The empty circles mark the onset of commensurate oscillations.

In Fig. 2a, we display the measured electron mobility (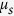) as a function of 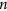. The mobility increases from 7.0 × 10^4^ cm^2^/V·s at 4.4 × 10^10^ cm^−2^ to 6.4 ×10^5^ cm^2^/V·s at 1.8 × 10^11^ cm^−2^. To characterize the degradation of mobility resulting from the nano-patterning steps, we plot in Fig. 2b the ratio of the mobilities of the superlattice sample and of an un-patterned sample (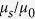). It can be seen that 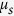 approaches 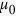 as 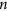 increases, and at 1.8 × 10^11^ cm^−2^, the degradation is only a factor of 2. In Fig. 2c we convert the mobility for the superlattice sample to mean free path. Over the density range, the mean free path increases from 170 nm to over 3 *μ*m.

### Commensurability oscillations

**Figure 3:**
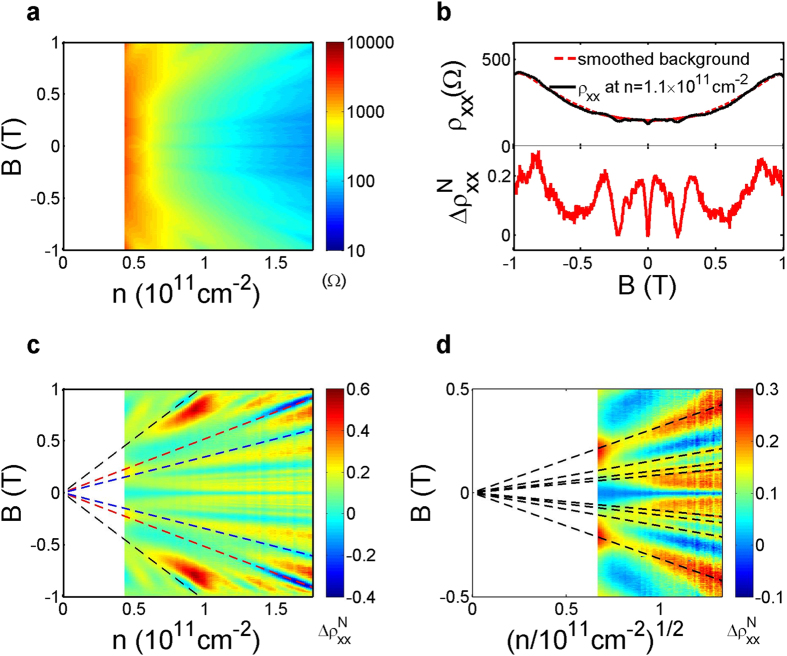
(**a**) Longitudinal resistivity 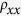 as a function of 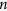 and 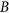 in logarithmic scale. (**b**) Upper panel: a line cut of 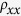 at *n* = 1.1 × 10^11^ cm^−2^ in linear scale is shown as the black line. Also plotted, as a red dashed line, is the smoothed background obtained by a 450-mT moving average filter. Lower panel: subtracting the smoothed background from 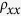 and normalizing to the zero magnetic field resistivity 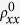 help visualize the oscillations over such a wide dynamic range in resistivity. (**c**) The same data as in (**a**) but with the background removed, normalized to 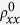, and plotted in linear scale. The black, red, and blue dashed lines represent, from left to right, the locations for Landau level filling factors *v* = 4, 8, and 12, respectively. (**d**) The same data as in (**c**), but with the x axis changed to 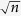 and a smaller range for the y axis. The linear black dashed lines are guides to the eye.

Our main result, the longitudinal resistivity 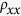 as a function of 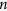 and 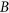, is plotted in Fig. 3a in logarithmic scale. As 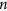 spans a fairly wide range, so does 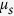, resulting in a dynamic range in 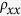 as wide as three orders of magnitude. To highlight the important oscillations we are interested in and to facilitate visualization of the data, we remove a slow-varying background along the 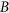 direction by a 450-mT-wide moving average filter and normalize the trace by 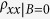 for each 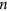. In Fig. 3b an example is shown for *n* = 1.1 × 10^11^ cm^−2^. The upper panel shows the original 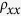 and the smoothed background, and the lower panel shows 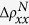, the normalized 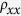 after removing the background. Figure 3c displays 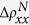, in linear scale. By comparing Fig. 3a,c it is clear that such data processing brings out features otherwise overwhelmed in the original plot. Also shown in Fig. 3c are three sets of dashed lines. The black, red, and blue lines represent the locations for Landau level filling factors *v* = 4, 8, and 12, respectively. It is from these Shubnikov-de Haas oscillations that we deduce 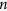.

We note that between −0.5 T < *B* < 0.5 T are a series of oscillations whose positions do not scale with 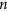 linearly, and thus are not consistent with Shubnikov-de Haas oscillations. In Fig. 3d we re-scale the x axis of Fig. 3c to 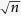 and focus on data at 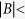 0.5 T. As can be seen in the plot, four sets of peaks symmetric with respect to 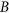 can be identified, with positions scaling linearly with 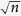, as indicated by the black dashed lines. We assign these peaks to commensuratbility oscillations, arising from cyclotron motion of electrons around one or more antidots.

### B-periodic quantum oscillations

**Figure 4:**
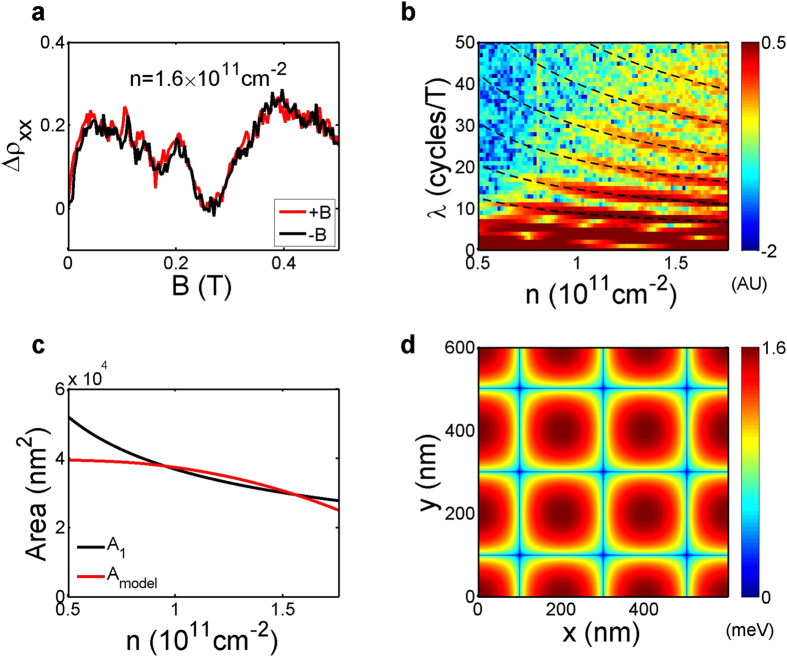
(**a**) 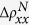 for *n* = 1.6 × 10^11^ cm^−2^ at low magnetic fields. (**b**) Amplitude of the Fourier spectra in logarithmic scale, obtained by Fourier transforming the data between *B* = −0.5 T and  = 0.5 T for every 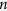. The black dashed lines are empirical fits of the form 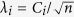. (**c**) Assuming the model potential is in the form of 
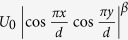
, we optimize 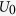 and 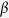 to obtain the best fitting between the deduced area 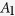 of the electron orbit encircling one antidot versus 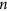, and the area 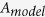 of a constant energy contour surrounding a single antidot at the Fermi energy. The obtained optimal 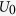 and 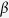 are 1.6 meV and 0.26. (**d**) The resulting model potential.

Zooming in the low-field 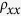, we can identify more oscillations symmetric with respect to 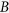 with smaller amplitudes, especially near the high-density end. We show one example in Fig. 4a for *n* = 1.6 × 10^11^ cm^−2^. To gain more insight, we Fourier transform along the 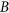 axis with 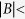 0.5 T to reveal 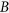-periodic oscillations. We plot in Fig. 4b the magnitude of the Fourier transform spectra in logarithmic scale, with the y-axis 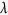 denoting the oscillation frequency per unit magnetic field (cycles/T). A series of peaks with increasingly high frequencies, indicated by black dashed lines, again sequentially emerge as 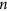 increases.

## Discussion

The induced 2D electrons in our superlattice device are of very high quality. Even after all the processing steps, the electron mobility remains on par with the best reported values for 2D electrons in modulation-doped Si/SiGe heterostructres^49^, which usually receive only minimal processing, i.e., ohmic contact metalization and annealing at a temperature around 400 °C, before electrical characterization. The mobility degradation is less than 10 over the entire density range, and approaches 2 on the high-density end. This is in sharp contrast to what is commonly observed for deep-etched modulation-doped samples^6^,^9^,^28^,^32^,^33^,^35^, and shows that keeping the integrity of the host heterostructure by avoiding etching, a novelty of our process flow, helps preserve the electron mobility. This wide density range allows us to probe regimes where the path length of an electron orbit is shorter than, comparable to, and longer than the zero-magnetic-field mean free path, and to observe a smooth evolution as 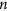 increases.

In a magnetic field, electrons execute cyclotron motions with a radius 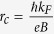, where 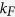 is the Fermi wavevector and 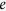 the elementary charge. A peak in magneto-resistivity is expected when the commensurability condition 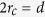, or more generally 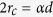, is met, where 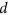 is the period of the superlattice and 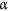 a constant determined by the specific shape of a periodic orbit^6^. Different peaks correspond to electron orbits with different radii encircling a certain number of antidots, or potentially a closed orbit between 4 antidots^27^. The resistance peaks can be understood in a simple semi-classical picture, where electrons executing periodic cyclotron motions do not contribute to conductivity and are removed from the phase space^6^. More sophisticated theoretical models show that it is the electrons performing chaotic motions near a periodic orbit in the phase space that are responsible for the resistivity peaks^50^. At any rate, the commensurability condition implies that the magnetic field at which a resistivity peak occurs is 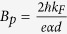, i.e., 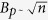, which is exactly what we observe in our sample, as shown in Fig. 3d. Deducing 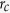 from 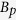 and 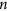 at each peak, we obtain radii of 116 ± 1, 231 ± 2, 340 ± 3, and 428 ± 1 nm for peaks in decreasing order of their magnetic field strength. These radii may represent orbits encircling 1, 4, 9 and 16 antidots, as shown by the red circles in Fig. 1(b). These numbers are consistent with previous observation^6^, with orbits encircling 2 and 10 antidots missing in our data. We note that the orbits that contribute to the magneto-resistivity are very sensitive to the exact superlattice potential^6^, and it is not surprising that we observe a different set of orbits. A striking feature of the data is the smooth evolution and sequential emergence of these peaks. To observe a resistivity peak due to one specific orbit, it requires that electrons do not experience scattering during one cycle of motion, or otherwise the effect would be destroyed. One metric we have for the scattering rate is the mean free path. The criteria for observing a resistivity peak is that the zero-magnet-field mean free path is longer than the orbit path length. We mark the circumference 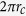 and the density at which a peak is first identifiable on the 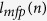 plot in Fig. 2c. The onset density of the last three peaks approximately follows 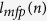, giving weight to this criteria. The discrepancy between this criteria and the onset of the first peak, representing electrons encircling a single antidot, is most likely caused by the presence of a resistance peak at low densities before the Landau level filling factor *v* = 8. The sequential emergence of commensurability oscillations demonstrates the strength of this device architecture, which allows for a wide, continuous, repeatable density range and its associated wide ranges of mobility and mean free path.

The sequential emergence of 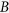-periodic oscillations with increasingly high frequencies, shown in Fig. 4b, is also enabled by the wide density range and high electron mobility in this unique device architecture. Starting from the peak with the lowest frequency, we denote the position of the *i*th peak as 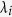. The 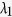 peak is weakly 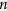-dependent and corresponds to approximately 10 cycles/T, or equivalently a period of 0.1 T. This period is interestingly very close to 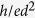. In fact, such 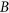-periodic oscillations with a period commensurate with the superlattice lattice constant have also been previously observed with 2D electrons in an antidot superlattice potential^27^,^28^,^30^,^31^,^51^, and are attributed to quantum oscillations of an electron orbit encircling one antidot^27^,^52^,^53^. We thus assign 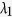 to the same physical origin. The other 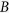-periodic peaks 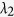 to 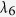 are associated with increasingly bigger closed orbits, and emerge sequentially as 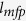 increases with 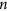. These higher-order oscillations have not been clearly observed in previous studies, presumably due to strong scattering in these more disordered samples^27^. These orbits, however, are sensitive to the superlattice potential due to their chaotic nature. It will require an accurate model of the potential and complicated numerical simulations to extract the exact paths of the orbits.

For an infinitely sharp, muffin-tin-like antidot superlattice potential, the shape of the constant energy contour at the Fermi energy is independent 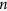, and so are the areas of closed electron orbits around one or multiple antidots. For a soft potential on the other hand, the area of the constant energy contour at the Fermi energy changes significantly as the Fermi energy varies, and so do the electron orbits. The density dependence of 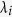 is thus a measure of the sharpness of the imposed superlattice potential, since the oscillation frequency in magnetic field is directly proportional to the orbit area. The six peaks observed here can be empirically fit by 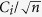, where 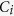 is a fitting parameter for 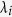. We obtain *C*_*i*_ = 2.81 ± 0.02, 4.62 ± 0.02, 6.86 ± 0.04, 9.49 ± 0.07, 12.64 ± 0.08, and 16.1 ± 0.2 × 10^6^/T·cm for *i* = 1–6, respectively. The empirical fitting curves are shown in Fig. 4b as black dashed lines. We note that the square-root dependence may be coincidental but provides a convenient single-parameter empirical description of our data.

Many previous theoretical studies on lateral square superlattices assumed a potential in the form of 
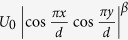
, where 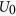 represents the strength the potential and 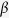 measures the sharpness^47^,^50^,^52^,^53^,^54^,^55^. To evaluate whether the superlattice potential in our device is considered sharp or soft, we assume a model superlattice potential in the same form, calculate the area 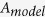 of a constant energy contour surrounding a single antidot at the Fermi energy as a function of 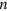 for given 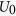 and 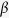. We optimize the fitting by minimizing the error between the calculated 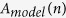 and 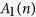, which is the area of the 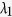 peak, obtained from 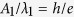. In Fig. 4c we plot 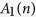 together with the optimal 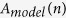. From the fitting we obtain *U*_0_ ~ 1.6 meV and *β* ~ 0.26. The resulting model potential is shown in Fig. 4d. As can be seen in Fig. 4c, the widely adopted model potential does not describe the actual potential very well, and thus any theoretical analysis based on a potential of this form should be done with caution. Nevertheless, the obtained 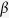 still qualitatively characterizes the softness of the superlattice potential. In the literature, *β* = 64 refers to a hard, muffin-tin-like potential, whereas *β* = 4 characterizes a soft superlattice potential. Our result is therefore far on the soft end. In our device the superlattice gate is separated from the 2DEG by a 30 nm-thick oxide layer and a 100 nm-thick SiGe barrier. Since the total thickness of the two intermediate layers is comparable to the diameter of the holes and the period of the superlattice, the potential modulation is expected to be soft in such Si quantum wells, as the numerical analysis suggests.

In summary, we present a novel process flow for making enhancement-mode heterostructure field-effect transistors with a lateral superlattice potential imposed on the capacitively induced 2DEG. The process flow preserves the quality of the underlying heterostructure, characterized by a small mobility reduction after patterning. The device architecture allows for a wide tunable density range, which enables our observation of sequential emergence of commensurability oscillations and quantum oscillations. From the density dependence of the quantum oscillations we are able to extract the sharpness of the superlattice potential directly.

Currently, two outstanding challenges in the area of conventional, laterally modulated 2D electrons are to observe definitive evidence of the Hofstadter’s butterfly^3^ and to make artificial graphene using semiconductor heterostructures^14^,^15^. Both tasks require a reasonably strong potential modulation and, perhaps more importantly, a relatively disorder-free starting 2D electron system. One approach toward artificial graphene, as was adopted by Gibertini *et al.*^11^ and Singha *et al.*^12^, is to etch modulation-doped starting material into tunnel-coupled quantum dots with a honeycomb superlattice. While interesting effects were indeed observed, the device showed low density and high resistivity after fabrication, and the linear energy-momentum dispersion remains elusive in transport experiments. An alternative approach is to make an antidot superlattice with triangular symmetry^10^,^13^. The process flow presented here is naturally compatible with this approach. Additionally, it was argued that such antidot superllatice may be more robust against disorder^16^. We thus believe that the architecture presented here may serve well as the platform for these experiments, and may enable new studies of band engineered materials with preserved host material quality and a wide tunable density range.

## Methods

### Material growth

The starting material used in this study was grown in a ultra-high-vacuum chemical-vapor-deposition system. The material stack consisted of a 10 Ω·cm p-type Si (100) substrate, a 1.4-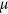
m SiGe graded buffer layer, a 3-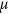
m relaxed SiGe buffer layer with Ge composition of 14%, a 20-nm strained Si layer, a 100-nm relaxed SiGe buffer layer with Ge composition of 14%, and a 2-nm Si cap. The strained Si layer serves as a quantum well for electrons in this heterostructure.

### Sample fabrication

Fabrication of the superlattice device started with ion implantation for ohmic contact formation. Phosphorus was implanted at 20 keV and 75 keV at a dose of 5 × 10^14^ cm^−2^ for both implant energies. The implanted dopants were activated by a rapid-thermal-anneal at 625 °C for 10 sec. The reduced thermal budget preserved the integrity of the strained Si quantum well^56^. Insulation between the gate and the ohmic contacts was achieved by depositing 30 nm of Al_2_O_3_ in an atomic-layer-deposition system. A blanket metal gate, consisting of 2-nm Ti and 40-nm Au, was then deposited. Ion milling was done to pattern the metal gate into a Hall bar. The implanted regions were contacted by locally etching away the insulator and depositing metal bond pads consisting of 2-nm Ti and 50-nm Au. The sample was then coated with PMMA with molecular weight of 950 K diluted in chlorobenzene to 4% for e-beam lithography. A hole array with a period of 200 nm in both directions was patterned in a rectangular area of 90 *μ*m × 180 *μ*m at the center of the device. Additional edge lines were also written to shrink the original Hall bar to a smaller one. After patterning, the sample was etched by ion milling to form the target superlattice with the e-beam resist acting as the etch mask. Etch stop was provided by the Al_2_O_3_ layer, as the selectivity of Au versus Al_2_O_3_ by ion milling is close to 20^57^.

### Measurement setup

Magneto-transport measurements were performed in a ^3^He cryostat with a base temperature of 0.3 K. All data were taken without illumination at the base temperature. Quasi d.c. measurements were done at 23 Hz. A constant bias of 1 mV_*rms*_ was supplied at one current lead, while the current through the device, the longitudinal voltage drop, and the transverse voltage drop were measured with standard lock-in techniques. The longitudinal resistance (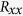), longitudinal resistivity (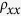), and the Hall resistance (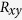) were then calculated. The gate voltage (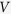) was swept unidirectionally at a fixed magnetic field (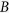), and 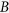 was incremented by 3 mT after each voltage scan.

## Additional Information

**How to cite this article**: Lu, T. M. *et al.* High-mobility capacitively-induced two-dimensional electrons in a lateral superlattice potential. *Sci. Rep.* **6**, 20967; doi: 10.1038/srep20967 (2016).

## Supplementary Material

Supplementary Information
