# Supplementary material for: High-mobility capacitively-induced two-dimensional electrons in a lateral superlattice potential
Source: Sci Rep. 2016 Feb 11;6:20967. doi: 10.1038/srep20967 (PMC4750089; doi:10.1038/srep20967)
Supplement: Supplementary Information [file srep20967-s1.pdf]

High-mobility capacitively-induced two-dimensional electrons in a lateral superlattice potential - supplementary information

T. M. Lu* and D. Laroche

Sandia National Laboratories, Albuquerque, New Mexico 87185, USA

S.-H. Huang, Y. Chuang, J.-Y. Li, and C. W. Liu

Department of Electrical Engineering and Graduate Institute of Electronic Engineering, National Taiwan University, Taipei 10617, Taiwan, R.O.C. and

National Nano Device Laboratories, Hsinchu 30077, Taiwan, R.O.C.

(Dated: January 6, 2016)

* tlu@sandia.gov

HALL RESISTANCE ANOMALY.

We extract the electron density from low-field R_{xy} (n') and also from high-field Shubnikov-de Haas oscillations (n). In Fig. S1a, we plot the ratio of n' over n. This ratio n'/n starts off higher than 1 on the low-density end, decreases as n increases, and appears to saturate at ~ 0.9. On the low-density end, the overestimation of n is due to suppression of R_{xy}. An example is given in Fig. S1b for $n = 4.7 \times 10^{10}\text{cm}^{-2}$, where a change in slope occurs at $|B| \sim 0.05$ T. On the high-density end, no change in slope is observed, but n' underestimates n. Fig. S1c shows an example for $n = 1.0 \times 10^{11}\text{cm}^{-2}$. The extrapolation of the low-field linear part of R_{xy} does not intercept quantum Hall plateaus at their centers, but goes over them.

For a simple 2D electron system the ratio n'/n is typically 1. The suppression of R_{xy} on the low-density end is similar to those previously reported for a 2D electron system in a lateral superlattice potential[1–4], which were attributed to chaotic motion of electrons in the antidot superlattice potential in the presence of a magnetic field[5]. In some cases, the suppression even leads to a change of sign of the Hall voltage[1]. The suppression we observe here is not as strong, and is quickly compensated by a competing mechanism as n increases. It becomes un-observable beyond $n = 6 \times 10^{10}\text{cm}^{-2}$, and instead n'/n drops below 1. One tentative explanation for the underestimation of n at high densities is that, in this density range where the mean free path is much longer than the superlattice period, n' measures the average density over the superlattice, while n probes a region of size comparable to the cyclotron radius and smaller than the superlattice period, and is thus minimally affected by the superlattice potential. In this case, the ratio n'/n represents the "area fill factor" of the superlattice region, *i.e.*, how much of each unit cell is filled by electrons, corrected by the suppression of R_{xy}. The area fill factor is expected to increase with n for a soft superlattice potential, which we demonstrate in the article is the case for our device. The saturation of n'/n thus requires the cancellation of the two correction mechanisms. It is unclear at the moment whether this cancellation is indeed the reason for the observed n-independent ratio at high electron densities.

REFERENCES

[1] Weiss, D. *et al.* Electron pinball and commensurate orbits in a periodic array of scatterers. *Phys. Rev. Lett.* **66**, 2790–2793 (1991).

[2] Schuster, R., Ensslin, K., Kotthaus, J., Holland, M. & Beaumont, S. Pinned and chaotic electron trajectories in an antidot lattice. *Superlattice Microst.* **12**, 93–96 (1992).

[3] Többen, D. *et al.* Electron transport through antidot superlattices in $Si/Si_{0.7}Ge_{0.3}$ heterostructures. *Phys. Rev. B* **50**, 8853–8856 (1994).

[4] Dorn, A. *et al.* Electronic properties of antidot lattices fabricated by atomic force lithography. *Appl. Phys. Lett.* **80**, 252–254 (2002).

[5] Fleischmann, R., Geisel, T. & Ketzmerick, R. Quenched and negative Hall effect in periodic media: Application to antidot superlattices. *Europhys. Lett.* **25**, 219–224 (1994).

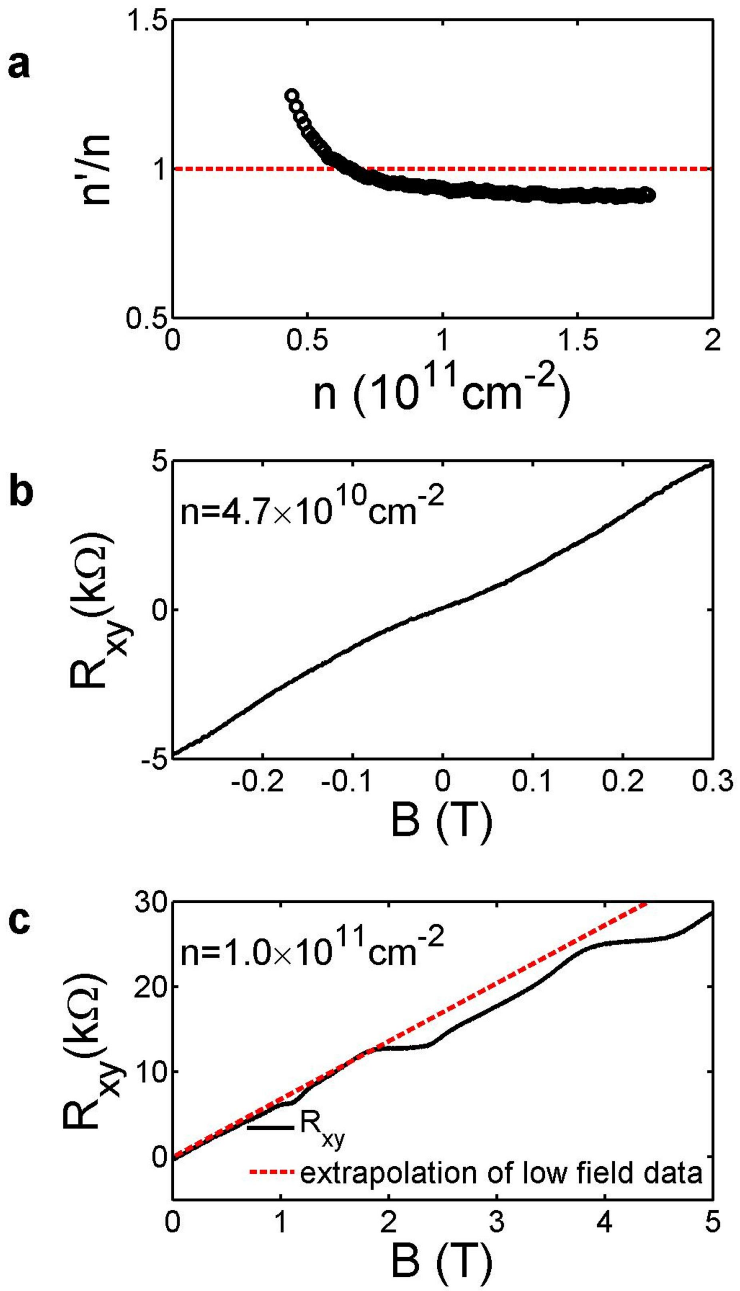

FIG. S1. (**a**) The ratio of n', determined from low-field R_{xy}, over n, determined from high-field Shubnikov-de Haas oscillations, as a function of n. The $n'/n = 1$ line is shown as the red dashed line. (**b**) Suppression of R_{xy} is observed at low densities. An example is shown here for $n = 4.7 \times 10^{10}\text{cm}^{-2}$. (**c**) At high densities, no suppression is observed, and instead the Hall coefficient extracted from low-field R_{xy} yields densities lower than n. An example is shown here for $n = 1.0 \times 10^{11}\text{cm}^{-2}$. The black curve is the measured R_{xy}, and the red dashed line is extrapolation of the low-field linear part.
